# WRAP53 is an independent prognostic factor in rectal cancer- a study of Swedish clinical trial of preoperative radiotherapy in rectal cancer patients

**DOI:** 10.1186/1471-2407-12-294

**Published:** 2012-07-17

**Authors:** Hong Zhang, Da-Wei Wang, Gunnar Adell, Xiao-Feng Sun

**Affiliations:** 1 Division of Biomedicine, The Systems Biology Research Center, University of Skövde, Skövde, Sweden; 2 Department of Stomatology, The Third Hospital of Hebei Medical University, Hebei, China; 3 Department of Oncology, Karolinska University Hospital, Karolinska, Sweden; 4 Division of Oncology, Department of Clinical and Experimental Medicine, Faculty of Heath Science, Linköping University, S-581 85, Linköping, Sweden

**Keywords:** WRAP53, Radiotherapy, Prognosis, Rectal cancer

## Abstract

**Background:**

Expression of WRAP53 protein has oncogenic properties and it is up regulated in several types of tumors.

**Methods:**

We examined expression of WRAP53 protein in rectal cancers and analyzed its relationship to the response to preoperative radiotherapy and patient survival. The WRAP53 protein was examined by immunohistochemistry in normal mucosa, primary tumors and lymph node metastases from 143 rectal cancer patients participated in a Swedish clinical trial of preoperative radiotherapy.

**Results:**

Frequency of WRAP53 protein expression was increased in primary rectal cancer compared to the normal mucosa (p < 0.05). In non-radiotherapy group positive WRAP53 in primary tumors (p = 0.03, RR, 3.73, 95% CI, 1.13-11.89) or metastases (p = 0.01, RR, 4.11, 95% CI, 1.25-13.14), was associated with poor prognosis independently of stages and differentiations. In radiotherapy group, positive WRAP53 in the metastasis correlated with better survival (p = 0.04). An interaction analysis showed that the correlations of WRAP53 with the prognostic significance with and without radiotherapy in the metastasis differed (p = 0.01). In the radiotherapy group, expression of WRAP53 in metastases gave a better outcome (p = 0.02, RR, 0.32, 95% CI, 0.13-0.84), and an interaction analysis showed significance between the two groups (p = 0.01).

**Conclusion:**

WRAP53 may be a new biomarker used to predict prognosis and to select suitable patients for preoperative radiotherapy.

## Background

Colorectal cancer is one of the most common types of cancer worldwide [1], and 50% of the patients still develop local or distant recurrence, and eventually die from colorectal cancer despite surgery [2]. In order to improve outcome of the cancer patients, several approaches, such as radiotherapy and chemotherapy, have been introduced to the treatmentof the cancer during the last decades. Preoperative radiotherapy has been proven to reduce local recurrence and further improve overall survival in rectal cancer patients [3-5]. However, there is marked difference in responses to the preoperative radiotherapy and prognosis between the cancer patients [6]. It is, therefore, a challenge to find valuable biomarkers for estimating different variations in the response to the preoperative radiotherapy and improving the patient prognosis.

*WRAP53* gene (for WD40-encoding RNA antisense to p53) encodes a regulatory RNA essential for p53 function upon DNA damage [7]. *WRAP53* also encodes a protein for maintenance of nuclear organelles called Cajal bodies [8-10]. WRAP53 protein has been found overexpressed in a broad range of human cancer cell lines in comparison to non-transformed cells. The WRAP53 overexpression promotes cellular transformation, whereas *WRAP53* knockdown triggers apoptosis of cancer cells [11]. Common variations in *WRAP53* (alias WDR79) have been associated with an increased risk for developing breast [12] and ovarian cancer [13]. However, there is no evidence concerning WRAP53 expression in rectal cancers, and its association with radiotherapy response.

In this study, we examined WRAP53 protein in biopsies, and surgical specimens from distant normal mucosa, adjacent normal mucosa, primary tumor and lymph node metastasis from the patients participated in a Swedish rectal cancer clinical trial of preoperative radiotherapy (Uppsala, 1986-11-17, Dnr. 86151) [4].

## Methods

### Rectal cancer patients

**Figure 1:**
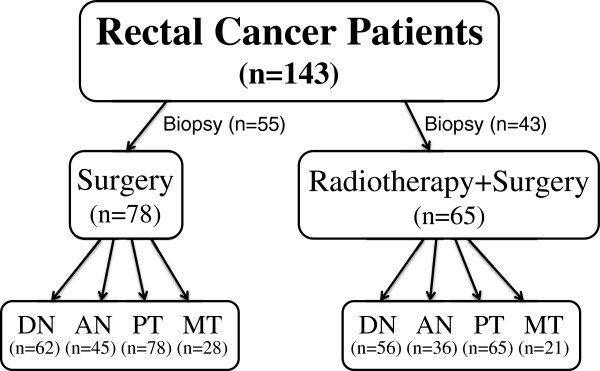
Profile of the rectal cancer patients including biopsies (taken before surgery and radiotherapy), and surgical samples of distant normal (DN), adjacent normal (AN), primary tumor (PT) and lymph node metastatic tumor (MT).

The sample profile of the rectal cancer patients showed in Figure 1. Biopsies (n = 98), distant normal mucosa (n = 118), adjacent normal mucosa (n = 81), primary tumors (n = 143) and lymph nodes metastases (n = 49) were from rectal cancer patients in the Southeast Swedish Health Care region, and they participated in the Swedish Rectal Cancer Clinical Trial of Preoperative Radiotherapy between 1987 and 1990, Radiotherapy, Uppsala, 1986-11-17, Dnr. 86151, [4]. There were 171 cancer patients who were randomised selected from Östergötland region, Sweden in the beginning. However, four patients were excluded due to surgically unresectable (advanced disease), and 24 patients had no available tissue specimen for this study. All patients had given their consent to participate in the study. The distant normal mucosa was histologically free from tumor taken from the distant margin, and 65 of them were matched with their primary tumors (i.e., from the same patients). Adjacent normal mucosa was adjacent to the primary tumor from the same tissue sections. Metastases were from the regional lymph nodes, and 37 of them were matched with their primary tumors. Seventy-eight of the patients received surgery alone and 65 received radiotherapy before surgery. The radiotherapy was given at a total of 25 Gy in 5 fractions before surgery over a median of 6 days (range, 5–12 days). Surgery was performed in a median of 3 days (range, 1–13 days) after radiotherapy. None of the patients received adjuvant chemotherapy before or after surgery, and all patients had locally resectable rectal adenocarcinoma. Mean age of the patients at diagnosis was 67 years (range, 36–86 years). All patients were followed-up, and the median of follow-up was 71 months (mean, 85 months). Other characteristics of the patients and tumors are present in Table 1.

**Table 1:** Characteristics of patients and rectal cancers

**Characteristics**	**Non-radiotherapy**		**Radiotherapy**	
	**No.**	**%**	**No.**	**%**
Gender				
Male	45	58	40	62
Female	33	42	25	38
Age (years)				
≤ 67	33	42	26	40
> 67	45	58	39	60
TNM stage				
I	21	27	18	28
IIA + IIIA + IIIB	39	50	38	58
IIIC + IV	18	23	9	14
Differentiation				
Well	5	6	4	6
Moderately	55	71	44	68
Poorly	18	23	17	26
Number of tumors				
Single	68	89	53	82
Multiple*	8	11	12	18
Surgical type				
Rectal amputation	42	54	25	38
Anterior resection	36	46	40	62
Rectal margin				
Tumor free	74	95	62	95
Tumor involved margin	4	5	3	5
Distance to anal verge (cm)				
Mean	7.3		8.6	

*Other colorectal cancer or other type of tumor besides the present rectal cancer.

The data for terminal deoxynucleotide transferase-mediated deoxyuridine triphosphate-biotin nick end-labeling (TUNEL) assay [14] and survivin expression by immunohistochemistry [15] were taken from previous studies performed in the same patients at our laboratory.

### Immunohistochemistry

Tissue arrays sections (5-μm) were deparaffinized, rehydrated and cooked (0.01M Tris-EDTA, pH 9.0) for 5 minutes, incubated in peroxides block (Dako, Carpinteria, CA) for 5 minutes and in protein block (Dako) for 10 minutes, the sections were incubated with rabbit α-WRAP53-C1 (WRAP53-483) polyclonal antibody [7] at 1:500 dilution at 4°C overnight, and then the secondary antibody EnVision/HRP, goat anti-rabbit (Dako), for 30 minutes. Generation of polyclonal α-WRAP53-C1 has been previously described [7]. The sections were then incubated by 3,3-diaminobenzidine tetra hydrochloride (Dako) for 8 minutes. Sections known to be positive for WRAP53 were included in each run, receiving either the primary antibody or PBS, as positive and negative controls.

Expression of WRAP53 protein was subjectively examined by two researchers (Zhang H and Wang DW). The investigators scored the sections in a blinded fashion without knowledge of clinicopathological data. The expression was scored as negative (<5%), weak, moderate and strong, and in statistical analyses, we divided cases with the staining into a negative (negative + weak staining) and a positive group (moderate + strong staining) due to the similarity of clinicopathological features in the cases with negative and weak staining versus the cases with moderate and strong staining.

### Statistical analysis

The differences in the frequency of WRAP53 expression and its association with clinicopathological/biological factors were analyzed by McNemar’s or Chi Square. The relationship between the WRAP53 and survival were analyzed by Cox’s proportional hazard model. The patient survivals were further analyzed by multivariate and interaction methods.

## Results

### WRAP53 Expression in rectal cancer

**Figure 2:**
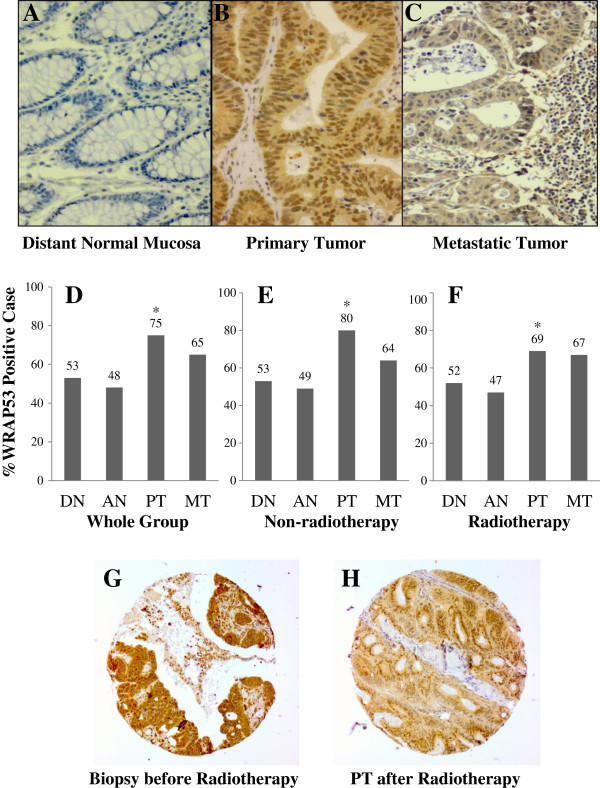
**Expression of WRAP53 protein in distant normal mucosa.** (**A**), primary tumor (**B**) and lymph node metastasis (**C**) from the same rectal cancer patient. Frequency of the WRAP53 expression in the whole group of rectal cancer patients (**D**), non-preoperative radiotherapy group (**E**) and preoperative radiotherapy group (**F**). Each bar represents the percentage of cases expressing WRAP53 in each subgroup: distant normal mucosa (DN), adjacent normal mucosa (AN), primary tumor (PT) and lymph node metastatic tumor (MT). The asterisks indicated the significant differences between the DN or AN and PT. The WRAP53 expression in a biopsy taken before radiotherapy (**G**) and in the corresponding primary tumor after radiotherapy from the same patient (**H**).

Immunohistochemistry was carried out in primary rectal cancers (n = 143), along with available distant (n = 118) and adjacent (n = 81) normal mucosa as well as metastasis in the lymph node (n = 49) to examine the expression and localization of the WRAP53 protein. Figure 2 shows expression of WRAP53 in rectal cancer and surrounding tissue from the same patient. The WRAP53 protein was localized both in the cytoplasm and the nucleus (Figure 2B, C).

In whole group of the patients, the WRAP53 expression was significantly increased from the distant (p < 0.0001) or adjacent mucosa (p < 0.0001) to the primary tumor (Figure 2A, B, D). Thus, the WRAP53 is overexpressed in primary rectal tumor compared to normal mucosa. However, the WRAP53 was not statistically different between primary and metastatic tumor (Figure 2D).

WRAP53 expression was further analyzed in patients who did not receive radiotherapy or the patients received radiotherapy. In the non-radiotherapy group, WRAP53 expression was significantly increased from the distant (p = 0.03) or adjacent mucosa (p = 0.03) to the primary tumor (Figure 2E). The WRAP53 expression was not statistically different between primary and metastatic tumor (Figure 2E). A significant increase of the WRAP53 was also found in primary tumors in the radiotherapy group compared to distant normal mucosa (p = 0.008) or adjacent normal mucosa (p = 0.01, Figure 2F). There was no difference between the primary tumor and metastasis in either of the groups (p > 0.05, Figure 2E, F). Thus, the primary rectal tumors show enhanced the WRAP53 expression compared to normal mucosa independently of radiotherapy.

We observed an increased number of WRAP53 positive cases in the non-radiotherapy group (80% WRAP53 positive, Figure 2E) compared to the radiotherapy group (69% WRAP53 positive, Figure 2F). To determine whether WRAP53 expression was changed after the radiotherapy, we analyzed the frequency of strong WRAP53 expression in biopsies removed before radiotherapy and in the corresponding primary tumors surgically removed after the radiotherapy. We found that expression of WRAP53 protein was decreased after radiotherapy: 45 biopsies (46%) expressed WRAP53 strongly compared to only 20 (31%) of primary tumors after radiotherapy (p = 0.05, data not shown). Expression of the WRAP53 was shown in the biopsy before radiotherapy (Figure 2G) and in the corresponding primary tumor after the radiotherapy (Figure 2H). There was no such evidence in comparison of the biopsies with the corresponding primary tumors without radiotherapy (77% Vs. 69%, p = 0.30). This finding suggests that expression of WRAP53 protein was down regulated in rectal cancers upon radiotherapy.

### WRAP53 Expression in relation to clinicopathological variables in rectal cancer patients

**Figure 3:**
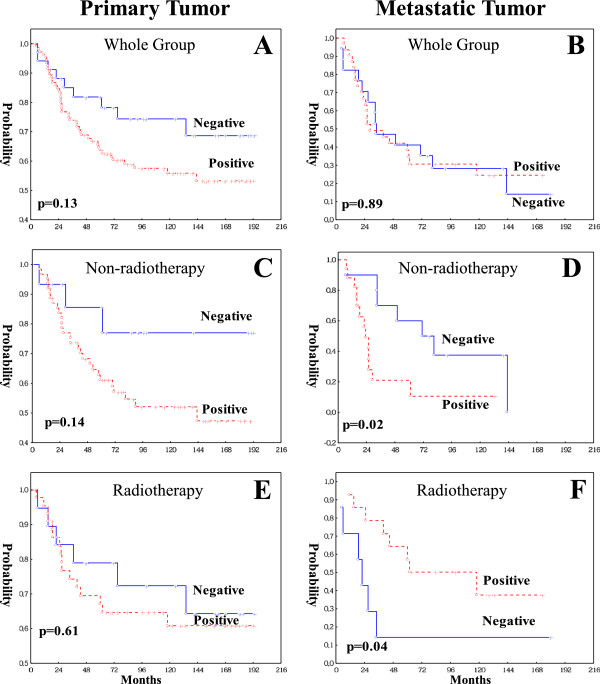
**Kaplan-Meier analysis (X-axis: survival time from the diagnosis date; Y-axis: **Patient overall survival probability) shows the correlation between WRAP53 expression and patients survival in 143 primary tumors **(A)** and 49 metastatic tumors in the whole group of the patients, non-radiotherapy group of 78 primary tumors **(C)** and 28 metastatic tumors **(D)**, as well as radiotherapy group of 65 primary tumors **(E)** and 21 metastatic tumors **(F)**.

In the whole group of patients, WRAP53 expression either in the primary tumors (p = 0.13, Figure 3A) or in the metastases (p = 0.89, Figure 3B) was not correlated to outcome of the patients or other clinical variables (p > 0.05, data not shown).

The relationship between the WRAP53 and patients survival was further examined in non-radiotherapy and the radiotherapy group. In non-radiotherapy group, WRAP53 expression in the primary tumors was associated with a worse prognosis in a multivariate analysis including both TNM stage and differentiation (p = 0.03, RR, 3.73, 95% CI, 1.13-11.89) although univariate analysis did not show a significant relationship (p = 0.14, Figure 3C). The patients with positive WRAP53 expression in the metastases had worse prognosis in the non-radiotherapy group in both univariate (p = 0.02, Figure 3D), and multivariate analysis including TNM stage and differentiation (p = 0.01, RR, 4.11, 95% CI, 1.25-13.14). However, there was no significant correlation between WRAP53 in the primary tumor and survival in the radiotherapy (Figure 3E). Surprisingly, in the metastases with radiotherapy, positive WRAP53 turned out to have better prognosis (p = 0.04, Figure 3F) although its prognostic significance was lost in a multivariate analysis including both TNM stage and differentiation (p = 0.14). A multivariate interaction analysis showed that the correlations with prognostic significance of WRAP53 expression in the metastases without and with radiotherapy differed significantly (p = 0.01). Thus, positive WRAP53 expression is a marker of worse prognosis for the patients without radiotherapy. However, in the radiotherapy group, positive WRAP53 expression in the metastasis showed the opposite correlation to survival and was thus more favorable for those patients.

**Figure 4:**
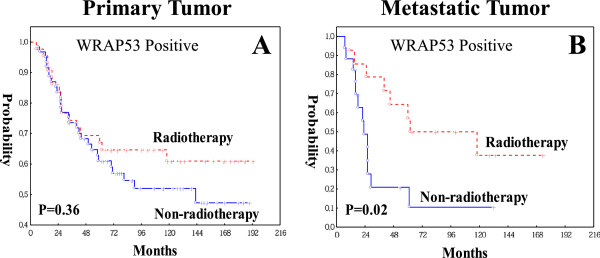
**Kaplan-Meier analysis (X-axis: survival time from the diagnosis date; Y-axis:** Patient overall survival probability) shows the correlation between preoperative radiotherapy and patient survival in the group of patients with positive expression of WRAP53 in 107 primary tumors **(A)** or in 32 metastatic tumors **(B)**.

We further analyzed the impact of radiotherapy on the patients survival based on WRAP53 expression. In WRAP53 negative group of either primary or metastatic tumors, radiotherapy had no prognostic effect (p > 0.05, data not shown). In the WRAP53 positive group, radiotherapy did not play a prognostic role in primary tumors (p = 0.36, Figure 4A). However, radiotherapy did give a better prognosis in the metastasis in either univariate (p = 0.02, Figure 4B) or multivariate analysis including both TNM stage and differentiation (p = 0.02, RR, 0.32, 95% CI, 0.13-0.84). A multivariate interaction analysis showed that the correlations with prognostic significance of radiotherapy differed significantly between the patients having positive WRAP53 and the patients with negative WRAP53 in the metastasis (p = 0.01). Thus, WRAP53 may be a novel predictive marker for response to the radiotherapy in patients with metastatic rectal cancer.

We also analyzed whether WRAP53 was related to clinicopathological factors in both the non-radiotherapy and radiotherapy group. In the non-radiotherapy group, among 18 patients having local recurrence, 94% showed WRAP53 positive primary tumors, while in 60 non-local recurrences, 75% had WRAP53 positive primary tumors (p = 0.07). In the radiotherapy group, among 5 patients with non-distant recurrence, 100% showed WRAP53 positive expression, while in 16 patients with distant recurrence, only 56% had positive WRAP53 (p = 0.07).

### WRAP53 In relation to apoptosis in rectal cancer

Based on the above results, especially the relationship of WRAP53 with radiotherapy and survival, we asked whether WRAP53 might function through an apoptotic pathway. We first analyzed whether WRAP53 was related to apoptosis and the survivin protein in the primary tumor samples (we had no available data of apoptosis and survivin expression from the lymph node metastasis).

In the radiotherapy group, the WRAP53 expression was positively related to apoptosis (p = 0.04) and negatively correlated to survivin (p = 0.002), namely, the tumors showing positive WRAP53 after radiotherapy, had a higher frequency of apoptosis and low frequency of surviving (data not shown). There was no such evidence in the non-radiotherapy (p > 0.05).

## Discussion

**Figure 5:**
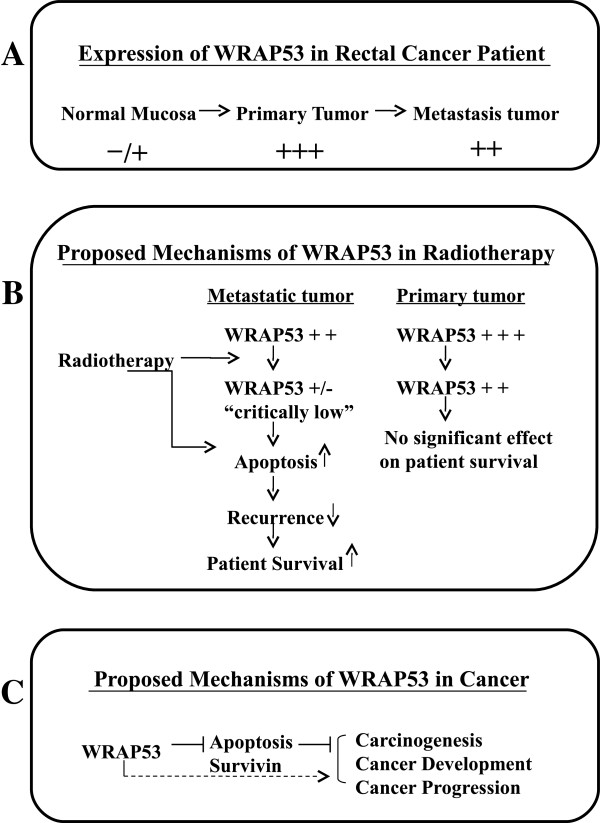
**Schematic illustration of WRAP53 in colon/rectal cancer.** In rectal cancer patients WRAP53 levels are significantly enhanced in primary tumor compared to normal mucosa, and then slightly decreased in metastatic tumors compared to primary tumors (**A**). Preoperative radiotherapy may inhibit the action of WRAP53 by reducing WRAP53 expression, subsequently increasing apoptotic activity or/and directly inducing apoptosis, leading to low local/distant recurrence, and eventually improve patients’ survival (**B**). In colon cancer cells WRAP53 blocks apoptosis and survivin protein, which are the defense mechanisms against tumor formation. This effect of WRAP53 may eventually leads to development and progression of a tumor. Alternatively, overexpression of WRAP53 could directly lead to malignant transformation of normal cells by other unknown mechanisms (**C**).

To our knowledge, this is the first report concerning WRAP53 expression and its association with prognosis in rectal cancer. We found that WRAP53 was increased from normal mucosa to primary rectal cancers. Considering previous reports on oncogenic properties of WRAP53 overexpression of WRAP53 contributes to malignant transformation [11], the enhanced WRAP53 in rectal cancer may be a sign of its involvement in the conversion of normal mucosa into cancer. We did not find statistical difference in WRAP53 expression between primary tumors and metastases, suggesting that the WRAP53 plays such role in the early stage of tumor development (Figure 5A). This exposes WRAP53 as a potential “oncoprotein” in early development of rectal cancer, and a new biomarker for early diagnosis of rectal cancer.

The patient material investigated here is from a Swedish clinical trial of preoperative radiotherapy in rectal cancer patients [4]. Therefore, the 143 rectal cancer samples were divided into two groups; tumors not treated with radiotherapy (n = 78) and tumors treated with radiotherapy (n = 65) in order to analyze the role of WRAP53 in the two groups. In the primary tumors without radiotherapy, positive WRAP53 was related to a higher frequency of local recurrence, and worse survival independently of stage and differentiation. A similar result was shown in the lymph node metastases without radiotherapy. This finding strengthens the role of WRAP53 in rectal cancer and identifies WRAP53 as a poor prognostic marker of primary and metastatic tumors without radiotherapy. Surprisingly, in the metastases with radiotherapy, the opposite relationship was observed. Positive expression of WRAP53 protein in this group turned out to have a lower frequency of distant metastasis and better survival. An interaction analysis in the two groups of the metastases with or without radiotherapy showed that the prognostic value of WRAP53 differed significantly. This correlation was only observed in the metastasis and not the primary tumors with radiotherapy. Moreover, radiotherapy only prolonged the patients’ survival in the metastases expressing WRAP53 but not in the metastasis lacking WRAP53. Could the slightly lower WRAP53 levels in the metastases compared to primary tumors in combination with radiotherapy be of importance? The tumors positive for the WRAP53 are addicted to WRAP53 and die by apoptosis upon removal of WRAP53 [11]. Could radiotherapy of the metastasis reduce WRAP53 to a “critically low level”, thus leading to induction of apoptosis? In such a case, it could explain why only the WRAP53 positive cells die upon radiotherapy, since they are addicted to WRAP53 expression. It could also explain why only the WRAP53 positive metastases die in response to radiotherapy, since they have less WRAP53 expression to start with and thus are more prone to reach a “critically low level” of WRAP53 required for induction of apoptosis (Figure 5B). Since primary tumors have a higher expression of WRAP53 protein the radiotherapy might not reduce the WRAP53 expression enough to induce WRAP53-specific apoptosis. It is well known that primary and metastatic tumors have different biological and clinical features. The numbers of genes distinguish metastases from primary tumors in colorectal cancer patients [16-19]. Even colon cancer cell lines, for example, KM12C, KM12SM and KM12L4a, with different metastatic potentials displayed different morphological and biological features after the treatments with radiation or drugs [20-22]. Thus, certain genes may be directly involved in primary tumor development whereas others play roles in metastasis. The weakness of this study is a small number of the patients in the each subgroup. It is necessary to confirm the results in a larger cohort of patients with rectal cancer with or without radiotherapy in the future. However, the results may raise a notion that we should not focus only on primary tumors but also on metastases in the identification of biomarkers when selecting patients for more efficient treatments.

The next question is whether the apoptosis pathway is involved in the association of WRAP53 with radiotherapy. Indeed, in rectal cancers with radiotherapy, there was a relationship of positive WRAP53 with increased apoptosis and decreased survivin. Moreover, WRAP53 positive colon cancer cells underwent spontaneous apoptosis upon reduction of WRAP53 expression. WRPA53 knockdown has been reported to result in a significant decrease in p53 mRNA and suppression of p53 induction upon DNA damage [7]. Notably, the survivin expression is negatively regulated by wild-type p53 in the p53-dependent apoptotic pathway [23,24]. As mentioned previously, in the strong expression of WRAP53 in biopsies was reduced in the primary tumors after radiotherapy. This provides a possible mechanism to the effects of radiotherapy on cancer. The curative effect of radiotherapy could partially be due to inactivation/down-regulation of WRAP53 protein, subsequently leading to cells apoptosis and necrosis. Alternatively, the radiotherapy and WRAP53 might have an additive effect to cause cell death (Figure 5B). WRAP53 expression has been connected to prognosis and radiotherapy in head-neck cancer [11]. In agreement with our study, high expression of WRAP53 was a marker for poor prognosis in head-neck cancer. Furthermore, the high levels of WRAP53 were correlated to radio-resistance of the head-neck cells. However, since no metastases of head-neck cancer were described it is difficult to compare our studies.

## Conclusions

WRAP53 protein may be a potential “oncoprotein” in rectal cancer development, and involved in induction of apoptosis in response to radiotherapy. We propose WRAP53 as biomarker for selecting suitable patients for preoperative radiotherapy.

## Competing interests

The authors declare that they have no competing interests.

## Pre-publication history

The pre-publication history for this paper can be accessed here:

http://www.biomedcentral.com/1471-2407/12/294/prepub
